# Symbolic Modeling of Asynchronous Neural Dynamics Reveals Potential Synchronous Roots for the Emergence of Awareness

**DOI:** 10.3389/fncom.2019.00001

**Published:** 2019-02-12

**Authors:** Pierre Bonzon

**Affiliations:** Department of Information Systems, Faculty of HEC, University of Lausanne, Lausanne, Switzerland

**Keywords:** symbolic modeling, neural dynamics, asynchronous process, synchronous process, emergence of awareness

## Abstract

A new computational framework implementing asynchronous neural dynamics is used to address the duality between synchronous vs. asynchronous processes, and their possible relation to conscious vs. unconscious behaviors. Extending previous results on modeling the first three levels of animal awareness, this formalism is used here to produce the execution traces of parallel threads that implement these models. Running simulations demonstrate how sensory stimuli associated with a population of excitatory neurons inhibit in turn other neural assemblies i.e., a kind of neuronal asynchronous wiring/unwiring process that is reflected in the progressive trimming of execution traces. Whereas, reactive behaviors relying on configural learning produce vanishing traces, the learning of a rule and its later application produce persistent traces revealing potential synchronous roots of animal awareness. In contrast, to previous formalisms that use analytical and/or statistical methods to search for patterns existing in a brain, this new framework proposes a tool for studying the emergence of brain structures that might be associated with higher level cognitive capabilities.

## Introduction

A recurring debate about the functioning of the brain concerns the characteristics and the roles played both at the neurological and cognitive levels by *synchronous* vs. *asynchronous* processes, their relation to *conscious* vs. *unconscious* behaviors, and a possible fundamental *duality* in neural dynamics. While the synchronous activation of brain processes is widely used for describing the functioning of the cortex (Singer, 1993), diverging views apply to the specialized tasks supported by these synchronized processes. Experimental results have revealed in particular the existence of transient long-range phase synchronization leading to the hypothesis that synchronization vs. desynchronization is a candidate mechanism for controlling visual attention (Gross et al., 2004). Other studies related to the integration of attributes in a visual scene suggest that there is no central neural clock involved in this mechanism, thus making the brain a massively asynchronous organ (Zeki, 2015). In support of this diversity, results from a large scale simulation (Markram et al., 2015) report “a spectrum of network states with a sharp transition from synchronous to asynchronous activity.” While no definite link between neural activity and conscious behavior (which would constitute *neural correlates of consciousness*) have been identified yet, it is common to postulate the existence of a dynamical *stream of consciousness* mediated by a global workspace (Baars, 1988) defined as a distributed brain state connected to various brain areas, thus making perceptual information available to different tasks. In one of these theories (Dehaene and Naccache, 2001) pertaining to the particular case of conscious perception (referred to also as *access consciousness*), sensory stimuli are associated with a population of excitatory neurons that in turn inhibits other neural assemblies, thus preventing the conscious processing of other stimuli.

More generally, an emergent picture of the brain shows opposing spiking patterns in populations of neurons engaged in a competition (Zagha et al., 2015). The demonstration of temporal competition in eligibility traces for long term potentiation and depreciation (*LTP/LTD*) designates these traces as plausible synaptic substrate for reward-based learning (He et al., 2015). Together, these findings enforce a fundamental principle in circuit neuroscience according to which inhibition in neuronal networks allows in turn for disinhibition and stands as a key mechanism for circuit plasticity, learning, and memory retrieval (Letzkus et al., 2015). Ideally, brain simulations should trace cognition down to these neurological. The usual way to simulate a brain today however still basically follows one of two bottom up approaches using either finite-state automata or differential equations. The first approach, which relies on weighted connections between neural cells to implement threshold logic without regard to the actual internal functioning of these cells, has led to the development of artificial neural networks (Hopfield, 1982; Hinton et al., 2006). These networks represent the most powerful tools available today in the field of *machine learning* and have been used to model circuits that reproduce human capabilities in pattern recognition. Their biological plausibility however is a subject of controversy, and their relevance to the study of the brain is thus questionable. The second approach simulates the electrical processes surrounding neurons, and thus details the functioning of the ground level constituents of real brains. So far these *neural networks simulators* (Hines and Carnevale, 1997; Markram et al., 2015) have not been applied to drive significant cognitive processes, but seem rather to expect and rely for that on the spontaneous emergence of higher level functions.

The “*what*” and “*how*” of *computational cognitive neuroscience* (Ashby and Helie, 2011) i.e., where computer and cognitive sciences meet in order to propose biologically plausible models supporting cognitive tasks, are traditionally described using the historical “tri-level” hypothesis (Marr, 1982) that distinguishes *computational, algorithmic*, and *implementation* levels. A fourth *behavioral learning* dimension in brain and cognition studies has been advocated (van der Velde and de Kamps, 2015) for by arguing that cognitive processes are executed in connection structures that link sensory circuits (i.e., perception) with motor (i.e., action). Bottom-up analytical tools such as differential equations, artificial neural networks as well as methods related to dynamical systems theory (Wright and Bourke, 2013), and more recently top-down approaches using abstract mathematical tools such Bayesian inference rules (Ma and Pouget, 2008), are well-suited for describing computations in Marr's sense, but “*fail to identify algorithms and underlying circuits”* (Frégnac and Bathellier, 2015), a task that calls for a “*middle-out*” approach that can identify plausible structures linking biology and cognition.

An assessment of the present situation in this field can be found in the special issue (Stern, 2017) of the *Science* journal entitled “*Neuroscience: In search for new concepts*,” which contains insightful reviews questioning present approaches, proposing conceptual challenges and asking neuroscientists to think about new ways to investigate them. Firstly, in order to identify the mechanisms which support the human brain, both Yarstev (2017) and Frégnac (2017) call for a *comparative approach* refining similar functions in specific behavior of relevant species where dynamic entities of simulated brains grow and interact with their environment. Next, as argued by Frégnac, “big data is not knowledge” i.e., the roadmap from data to knowledge should be mapped out across successive levels of integration distinguishing *micro-scale* and *meso-scale* functions. The causal link between sub-cellular/cellular mechanisms and behavior should be achieved through successive levels of analysis, as exemplified by Marr's *tri-level hypothesis*, which means that mappings need to be expressed in *algorithmic* terms and not just in a correlative way. In order to take into account intermediate levels of circuit integration, canonical operations should be defined as *invariant* computations. Furthermore, simulations elaborated from static atlases, or connectomes, are not sufficient to model brain functions where neurons participate in multiple functional sub-networks. Toward this end, Frégnac eventually suggests that a formalism based on virtual free “*quasi-particles*” may simplify the analytical treatment. Finally, Buzsaki and Llinas (2017) note that the neuronal mechanisms associated with navigation and memory are similar, meaning they process *messages* regardless of their origin. Toward this goal, a new approach (Bonzon, 2017) to modeling neural dynamics that enforces the tri-level framework based on synaptic plasticity illustrated in Frégnac (2017) has been proposed. In order to handle messages, synaptic plasticity is abstracted through *asynchronous communication* protocols and used to link perception and action. This formalism is used here to address the duality between synchronous vs. asynchronous processes, and their possible relation to conscious vs. unconscious behaviors.

## Materials and Methods

### A New Approach to Modeling Abstract Brain Functionalities

A new approach to modeling neural dynamics (Bonzon, 2017) that enforces the tri-level framework based on synaptic plasticity illustrated in Frégnac (2017) has been proposed. In order to handle messages, synaptic plasticity is abstracted through *asynchronous communication* protocols and used to link perception and action. This has been illustrated (Bonzon, 2017) though the simulation of simple animal behaviors. In this formalism, brain processes are first abstracted through virtual *microcircuits* representing canonical *invariant* computations as called for above. Sets of microcircuits are then assembled into *mesoscale* virtual circuits linking perceptions and actions. *Virtual circuits* giving rise to streams can be compiled into *virtual code implications* to be eventually used just in time to deduce *virtual code instructions* that are finally interpreted by a virtual machine (see the Supplementary Information section).

While the usual approach to simulating neural dynamics starts with current flows represented by differential equations, we opted for a conceptual abstraction of synaptic plasticity represented by communicating processes between concurrent *threads*, which correspond either to a single or to a group of neurons possibly interleaved at a higher level. Contrary to traditional neuron models in which incoming signals are summed in some integrated value, thread inputs can be processed individually, thus allowing for threads to maintain parallel asynchronous communications. Threads can be grouped into disjoint sets, or *fibers*, to model neural assemblies (Huyck and Passmore, 2013), and discrete *weights* (e.g., integer numbers) can be attached to pairs of threads that communicate within the same fiber. A fiber containing at least one active thread constitutes a *stream*. On this basis, a short term cache memory (*STM*) as well as a long term associative memory (*LTM)* relying on *LTP/LTD* were defined. This eventually led to the modeling (Bonzon, 2017) of animal behaviors exhibiting, among others, rule learning capabilities (Zentall et al., 1981; Katz et al., 2008) demonstrating primitive forms of animal consciousness according a typology (Pepperberg and Lynn, 2000) proposed in the context of comparative zoology.

### Basic Concepts

**Figure 1:**
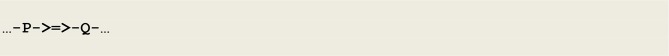
Circuit fragment implementing a synaptic transmission.

To introduce the basic concepts of this formalism, let us consider a simple case of synaptic transmission between any two threads **P** and **Q** (*NB throughout this text, identifiers starting with a capital letter stand for variable parameters*). This can be represented by the circuit fragment (or wiring diagram) contained in the simple stream given in Figure 1, where the symbol **- > = > -** represents a synapse.

**Figure 2:**
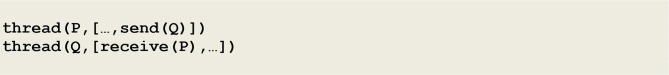
Thread patterns for a synaptic transmission.

This circuit fragment can be represented by two symbolic expressions involving a pair of **send/receive** processes as shown in Figure 2.

**Figure 3:**
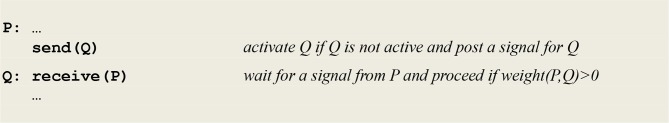
Communication protocol for an asynchronous communication.

In Figure 2, the thread **P** e.g., a sensor thread **sense(s)**, will fire in reaction to the capture of an external stimulus **s**, with the **send** process corresponding to the signal, or spike train, carried by a pre-synaptic neuron's axon. In the thread **Q** [e.g., an effector thread **motor(X)**, where the variable **X** becomes instantiated as the result of the stimulus], the **receive** process represents the possible reception of this signal by a post-synaptic neuron. The compilation of these expressions will give rise to virtual code implications implementing the communication protocol given in Figure 3.

This protocol corresponds to an *asynchronous* blocking communication subject to a threshold. It involves a predefined weight between the sender **P** and the receiver **Q** that can be either incremented or decremented. On one side, thread **P** fires thread **Q** if necessary and sends it a signal. On the other side, thread **Q** waits for the reception of a signal from thread **P** and proceeds only if the weight between **P** and **Q** stands above a given threshold. The overall process amounts to opening a temporary *pathway* between **P** and **Q** and allows for passing data by instantiating variable parameters (see example below).

### A Simple Model of Classical Conditioning

**Figure 4:**
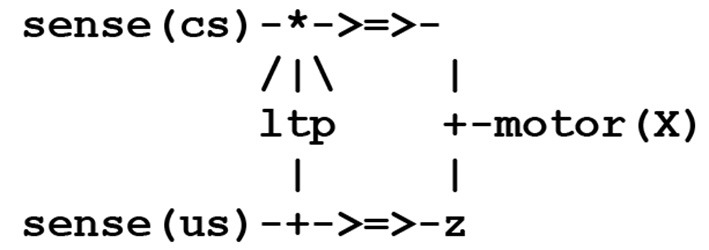
A mesoscale virtual circuit implementing classical conditioning.

As an example, let us consider a simple model of classical conditioning in which a conditioned stimulus **cs** elicits a weak reflex, and a unconditioned stimulus **us** produces a massive reflex. After a few pairings of **cs** and **us**, where **cs** slightly precedes **us**, a stimulus **cs** alone triggers an enhanced reflex. This is represented by the virtual circuit given in Figure 4.

**Figure 5:**
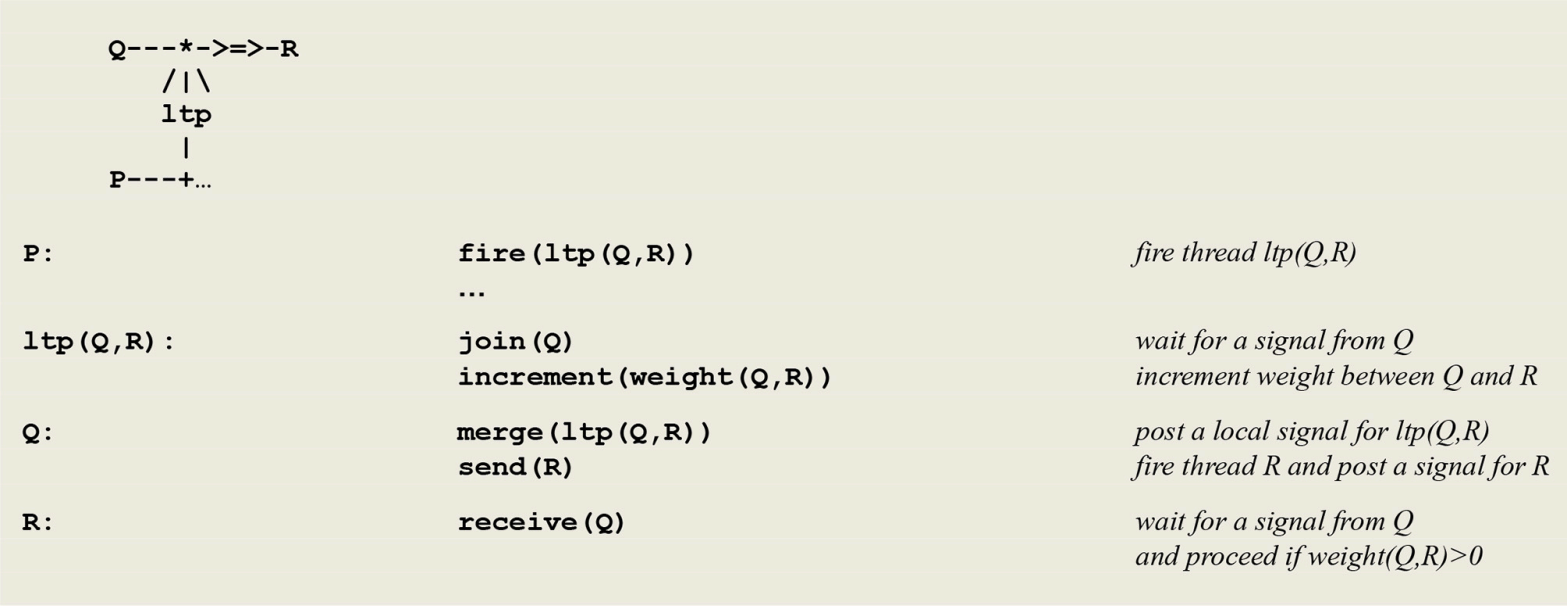
Micro-circuit and communication protocol for *ltp*.

In Figure 4, the threads **sense(us)** and **sense(cs)** correspond to sensory neurons, and **motor(X)** to a motor neuron, where **X** is a variable that will be instantiated into **us** or **cs**. Finally, the thread **ltp** (for *long term potentiation*) acts as a facilitatory interneuron reinforcing the pathway (i.e., augmenting its *weight*) between **sense(cs)** and **motor(cs)**. The protocols depicted by the symbols **- > = > -** and **/|\** represent, respectively, a *synapse* and the modulation of a synapse, the sign ^*^ indicates the conjunction of converging signals, and the sign + either the splitting of a diverging signal, as used in the lower branch, or, a choice between converging signals, as used in the right branch instantiating the thread **motor(X)**. Classical conditioning then follows from hebbian learning i.e., “neurons that fire together wire together.” Though it is admitted today that classical conditioning in aplysia is mediated by multiple neuronal mechanisms including a post-synaptic retroaction on a presynaptic site, the important issue is that the learning of a new behavior requires a conjoint activity of multiple neurons that leads to implement the thread **ltp** as a *detector of coincidence*, as done in Figure 5.

The generic microcircuit abstracting the mechanism of long term potentiation *(***ltp**) is given in Figure 5. In order to detect the coincidence of **P** and **Q**, thread **P** fires an **ltp** thread that in turn calls on process **join** to wait for a signal from thread **Q**. In parallel, thread **Q** calls on process **merge** to post a signal for **ltp** and then executes a **send(R)** command to establish a link with thread **R**. After its synchronization with thread **Q**, thread **ltp** increments the weight between **Q** and **R**.

### A Model of a Simple Case of Operant Conditioning

**Figure 6:**
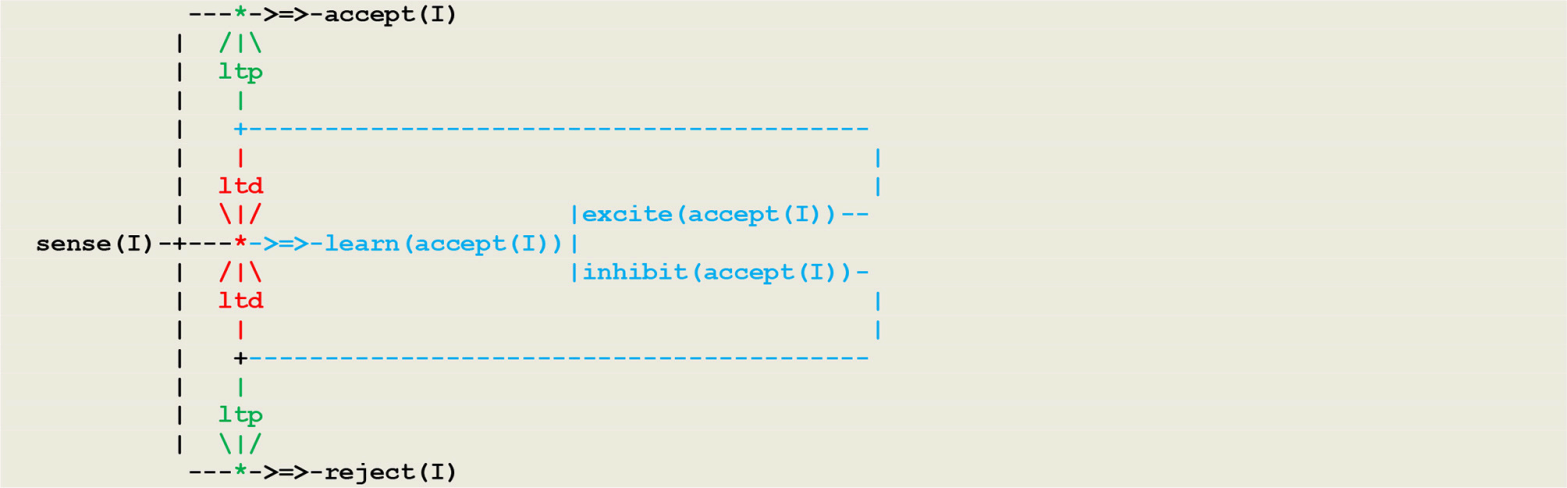
A virtual circuit implementing simple operant conditioning.

As another example, let us consider a simple form of *operant conditioning* in which an organism, as a result of a perception, generates either an excite or an inhibit internal stimulus and associates this feedback with either an accept or reject action. This gives rise to two competing neural populations, as represented in the circuit given in Figure 6 in which inputs are represented by a vector *I* of external perceptions.

At the beginning of the simulation, the pathways from **sense(I)** to learn[accept(I)] is open, while the pathways to both accept(I) and reject(I) are closed. After a few trials, the pigeon will have learned to close learn[accept(I)] through an **ltd** process and to open either accept(I) or reject(I)through an ltp process. This procedure matches a fundamental principle in circuit neuroscience according to which *inhibition* in neuronal networks during baseline conditions allows in turn for *disinhibition*, which then stands as a key mechanism for circuit plasticity, learning, and memory retrieval (Letzkus et al., 2015).

### Communication Protocols

As illustrated and briefly discussed above, virtual circuits rely on communication protocols that are pictured in thread diagrams by iconic symbols representing themselves microcircuits. These protocols are defined by pairs of procedures:

-**send/receive**, denoted by the symbols **- > = > -** or **-<=<-**, represents a synaptic transmission
-**join/merge**, denoted by **/|\** or **\|/**, implements long term potentiation/depression (**ltp/ltd**)
-**push/pull**, denoted by -<A>-, models a short term cache memory (**stm**)
-**store/retrieve**, denoted by -{P}-, models an associative memory (**ltm**) based on long term storage and retrieval (**lts/ltr**)

The microcircuits implementing these protocols are detailed in Bonzon (2017).

### Virtual Machine Definition

**Figure 7:**
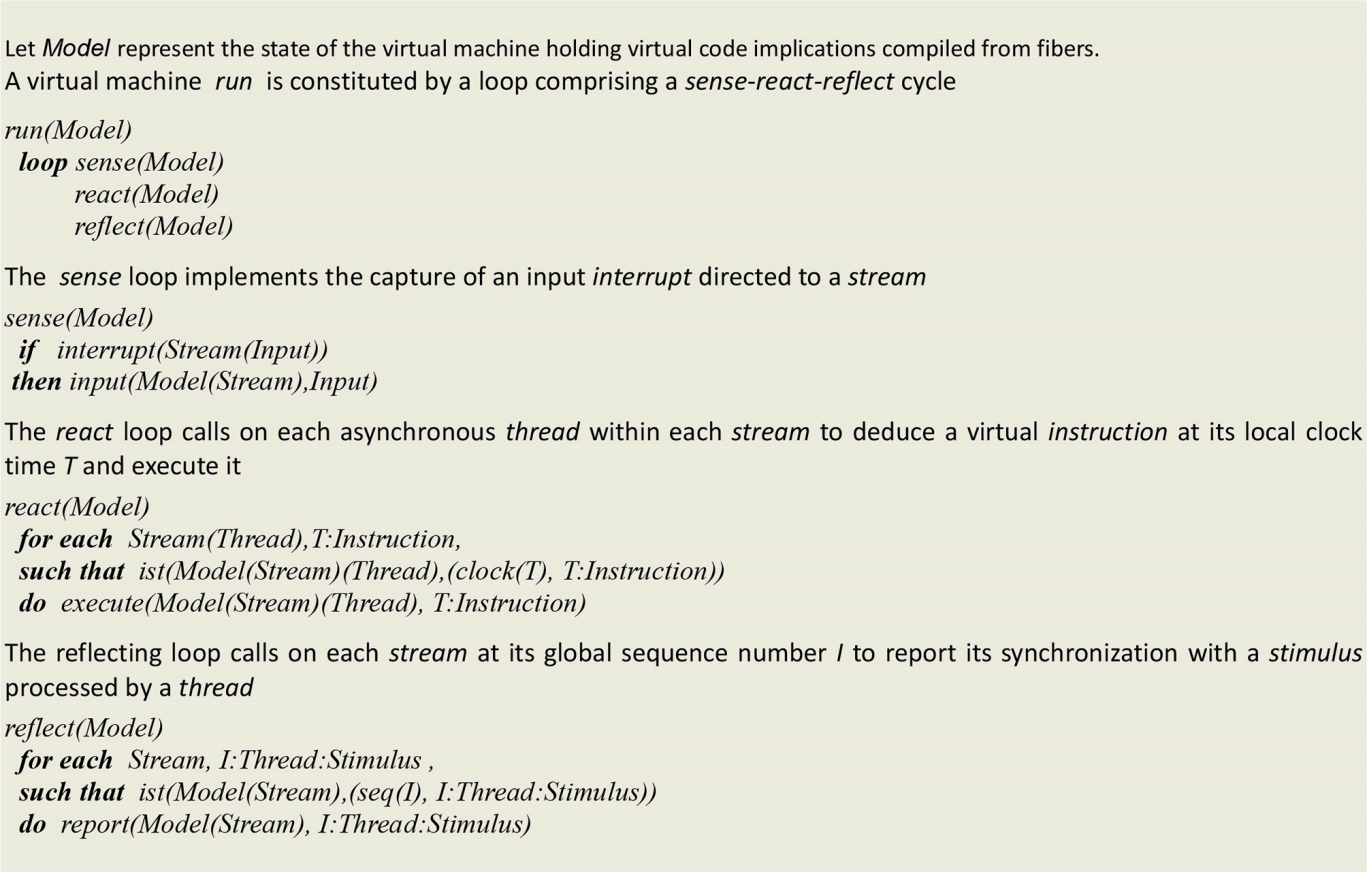
High level definition of a virtual machine run.

The virtual machine (Bonzon, 2017), which was originally designed to execute a “*sense-act*” cycle of embodied cognition, is extended here to implement a “*sense-act-reflect*” cycle that allows for tracing down the sequences of *synchronized* events associating a thread and a stimulus (see Figure 7 for the functional definition of this machine, and the online Supplementary Information for its complete operational specifications).

Let us just mention some characteristics of this machine that clearly distinguish it from traditional computers of the von Neumann type. First, it does not involve stored program acting on stored data. Consequently, this machine doesn't have an instruction register holding the current instruction being executed after its retrieval from an addressable memory. The *ist* predicate (standing for “is true”) implements *contextual deduction* (Bonzon, 1997). A register *clock (T)*, which corresponds to a program counter in traditional machines, is associated with each thread and hold its local time *T*. These registers are used in turn to deduce an instruction. Whenever an instruction succeeds, its thread clock is advanced and the next instruction is deduced, and whenever it fails, it is executed again until it eventually succeeds. Altogether, this amounts to descending into a thread instruction tree, with its local clock time corresponding to the currently reached depth. In other words, as postulated for instance by Zeki (2015), there is no central clock, thus “making of the brain a massively asynchronous organ.” The execution of virtual instructions leads to a wiring/unwiring process that produces model configurations that are akin to plastic brain states. By interpreting code deduced configurations that are akin to brain states, the overall architecture of this system could thus turn out to be close to that of a brain.

The core of a simulation platform implementing the formalism described above is defined by a logic program of about 300 lines. This platform can be run on any PC equipped with a Prolog compiler, which thus allows for an easy reproduction of results.

## Results

### Simulating Simple Animal Behaviors

In order to explore a possible duality between synchronous vs. asynchronous processes and conscious vs. unconscious behaviors, we used our extended formalism to perform a series of simulation of simple animal behaviors exhibiting in turn the first three level of animal consciousness according to Pepperberg and Lynn's typology (Pepperberg and Lynn, 2000). While this taxonomy is not the definite source on the subject, their proposal does comply with the requirements listed in our Introduction i.e.,

- they follow a *comparative approach* refining similar functions in the behavior of relevant species
- they include an *evolutive learning* dimension
- they can be implemented by canonical operations defined as *invariant* computations that constitute particular cases of *operant conditioning* linked to plausible neural processes.

Briefly, the first level of animal awareness corresponds to the ability to follow a simple rule involving the perception of a specific item or event and then either its acceptation or its rejection (e.g., a case of *matching/oddity* to sample). Whereas, this first level does not allow for an immediate transfer to a similar task, an organism with the second level is aware enough of a rule to transfer it across situations and thus to adopt for example a *win/stay lose/shift* rule (or strategy) relying on a short-term memory. In order to make a categorical judgment (e.g., to *sort* items by recalling their properties), the third level of animal awareness provides an organism with the additional capacity to integrate two different sets of stored information. This implies in turn some kind of associative long term memory.

#### A Simulation of the First Level of Animal Awareness

**Figure 8:**
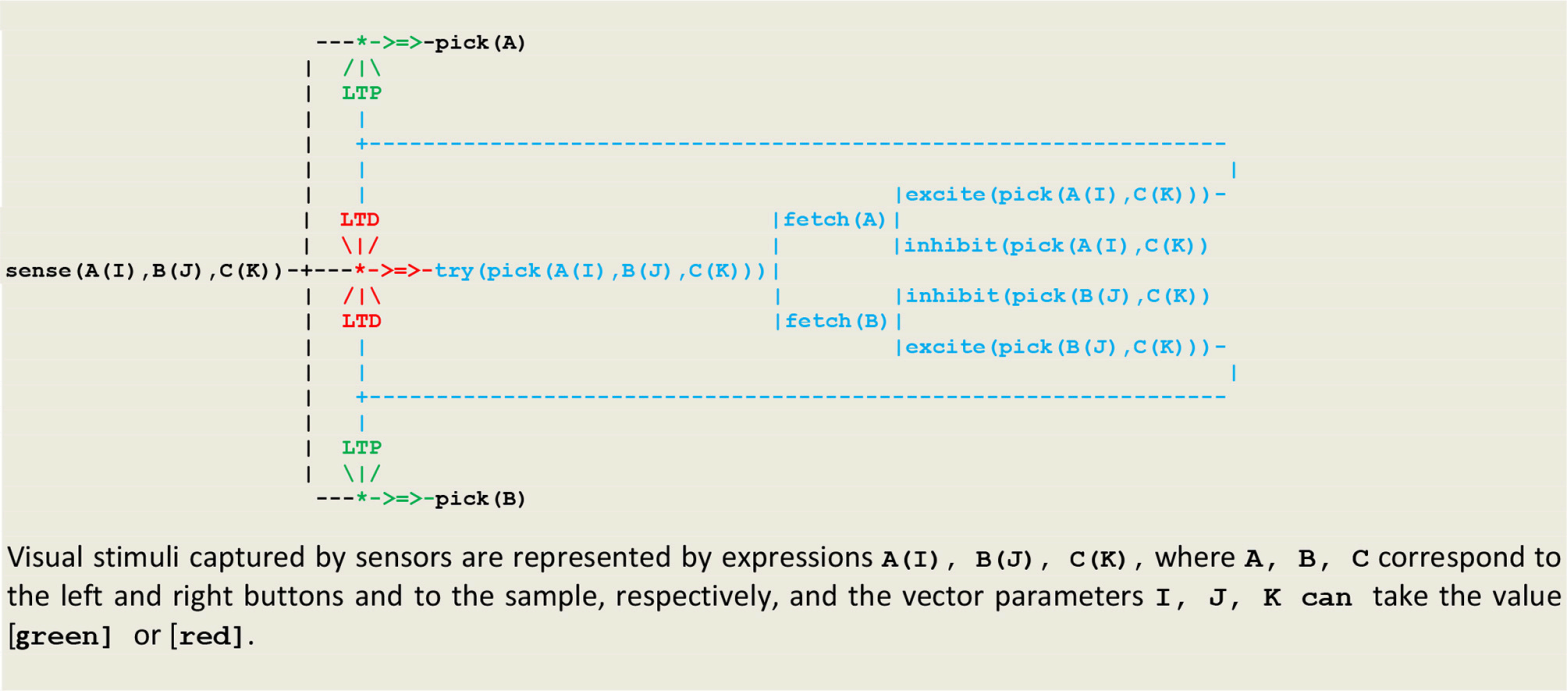
Circuit for configural learning.

Our first simulation refers to an experiment (Wright, 1997) that was designed in order to discriminate between two possible strategies for solving a *non-matching-to sample* (*NMTS*) task (Katz et al., 2008). In this experiment, a subject (e.g., a pigeon) is presented with a sample that can be of one of two colors (e.g., red or green), and then confronted with a pair of buttons (e.g., one left and one right button) of two different colors, one of them matching the color of the sample. In order to get a reward, the subject must choose the button that doesn't have the same color as the sample. A first strategy, called *configural learning*, is to learn the correct choice associated with each combination of colors (or external stimuli). The resulting unconscious reactive behavior then relies on memorized *links* between perception and action. This strategy is implemented in the circuit given in Figure 8 that constitutes an extension of Figure 6 including an internal fetch stimulus that triggers a random choice between the two buttons.

**Figure 9:**
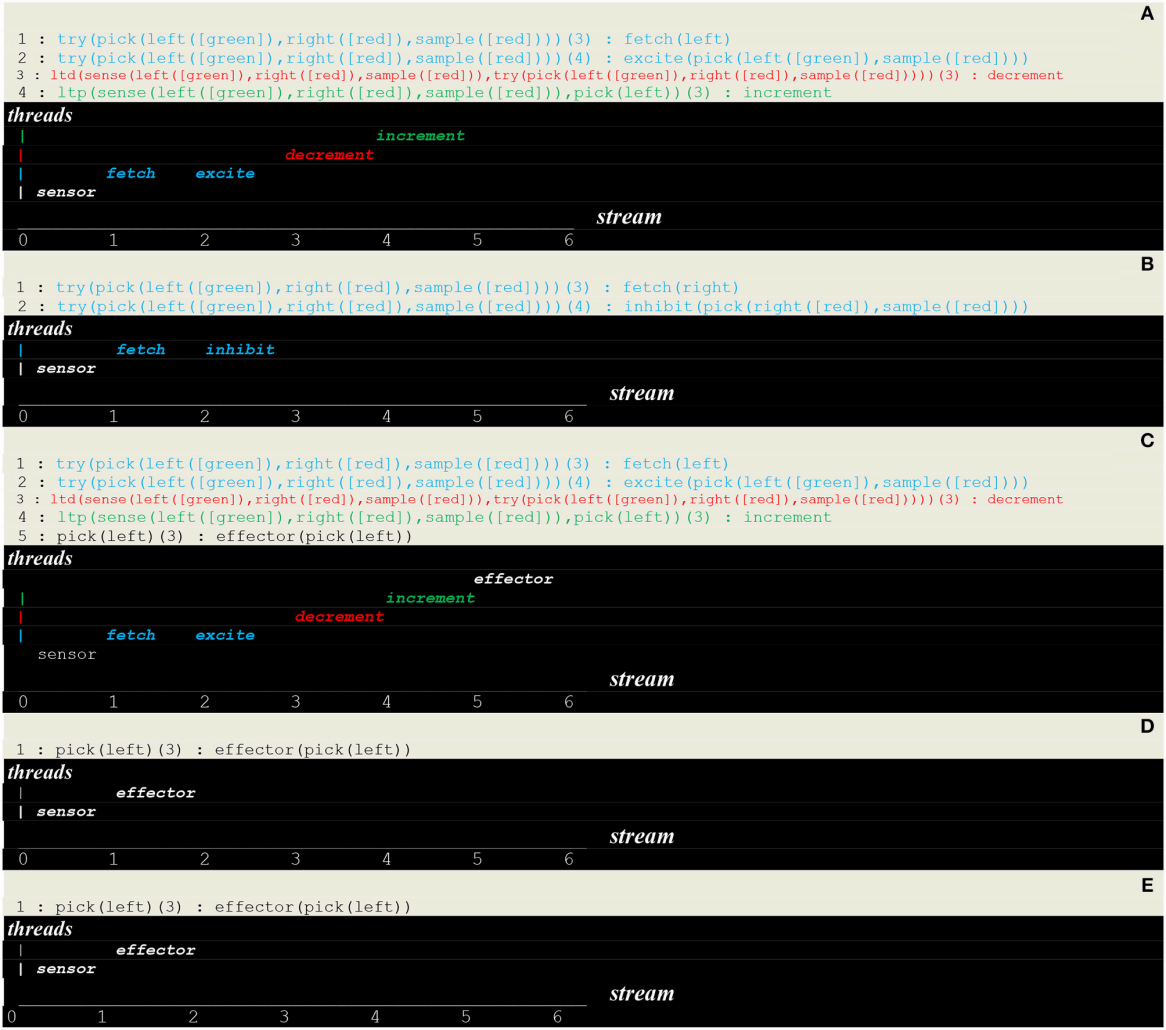
Execution trace. **(A–C)** Transient part. **(D,E)** Void part.

The execution trace of a running simulation is given in Figure 9. In this example, the same vector i.e., **[sensor(left([green]),right([red]),sample([red]))]** was repeatedly presented as input. The prefixes **1:, 2:, 3**:, etc., represent the stream's sequence numbers ***I*** akin to a global time series and the arguments (3), (4), (3), (3), etc., are threads local times. These traces contain first a transient part (Figures 9A–C) corresponding to the learning process. This process is implemented via successive internal fetch and excite/inhibit stimuli that give rise in turn to the increment/decrement of synaptic weights. This demonstrates how internal stimuli associated with a population of excitatory neurons inhibit in turn other neural assemblies i.e., a kind of neuronal wiring/unwiring process that is reflected in the progressive trimming of the execution trace. The second part (Figures 9D,E), void of any internal stimulus, then reflects an unconscious reactive behavior associating a *sensor* and an *effector*.

**Figure 10:**
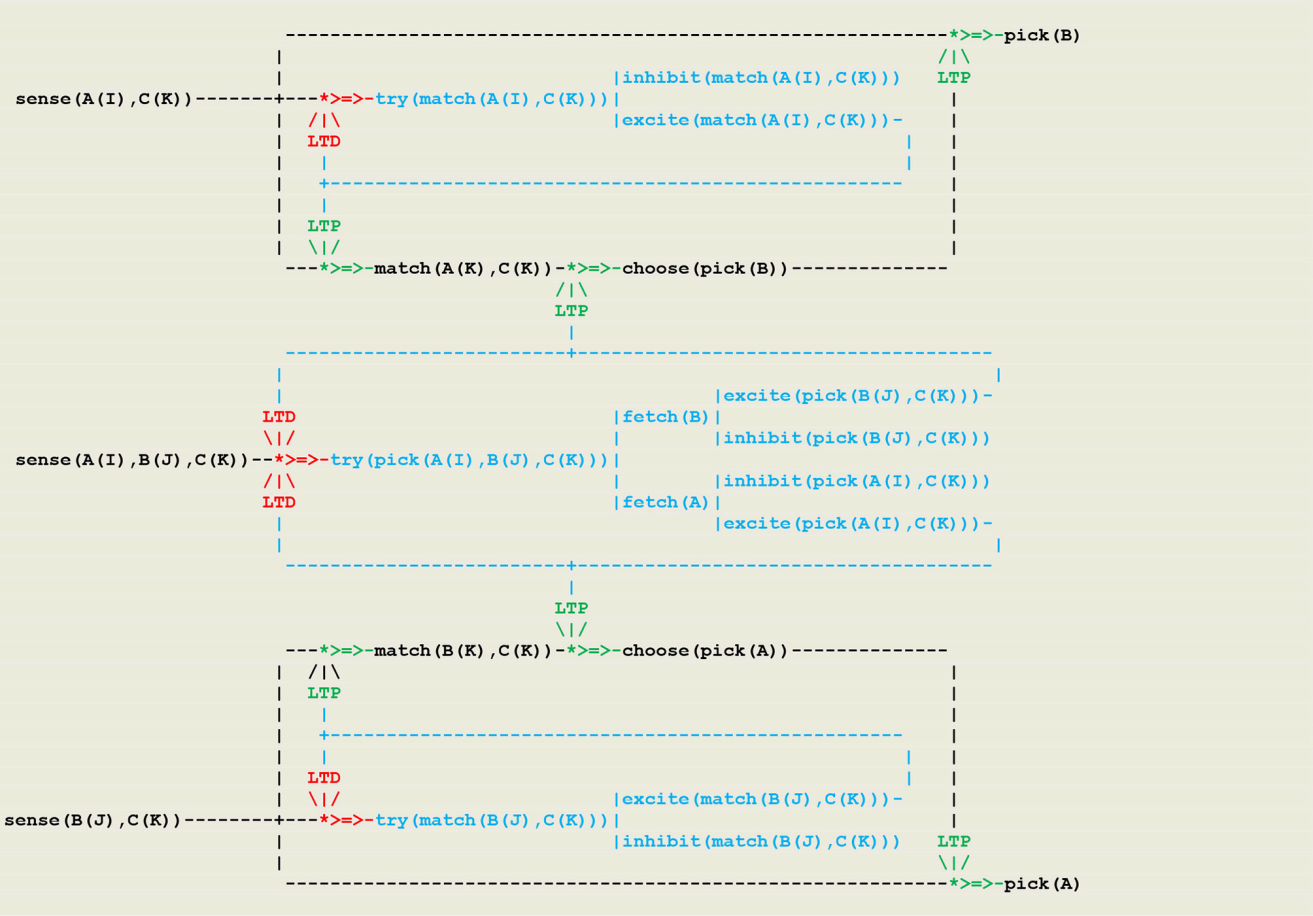
Circuit for rule learning.

Another strategy, called *relational learning*, is to compare in turn each button with the sample, learn to match colors, and then choose the button that doesn't match the sample. In other words, subjects do not learn to choose a color, but to match colors and then avoid the matching color i.e., to choose the non-match. This behavior corresponds to the first *level* of animal consciousness defined as the ability to learn and apply a simple *rule* associating the perception of a specific concept or event and then either its acceptation or its rejection (Pepperberg and Lynn, 2000). This strategy is implemented in the circuit given in Figure 10, where two additional layers implement learning to match and eventually choosing to avoid the match.

**Figure 11:**
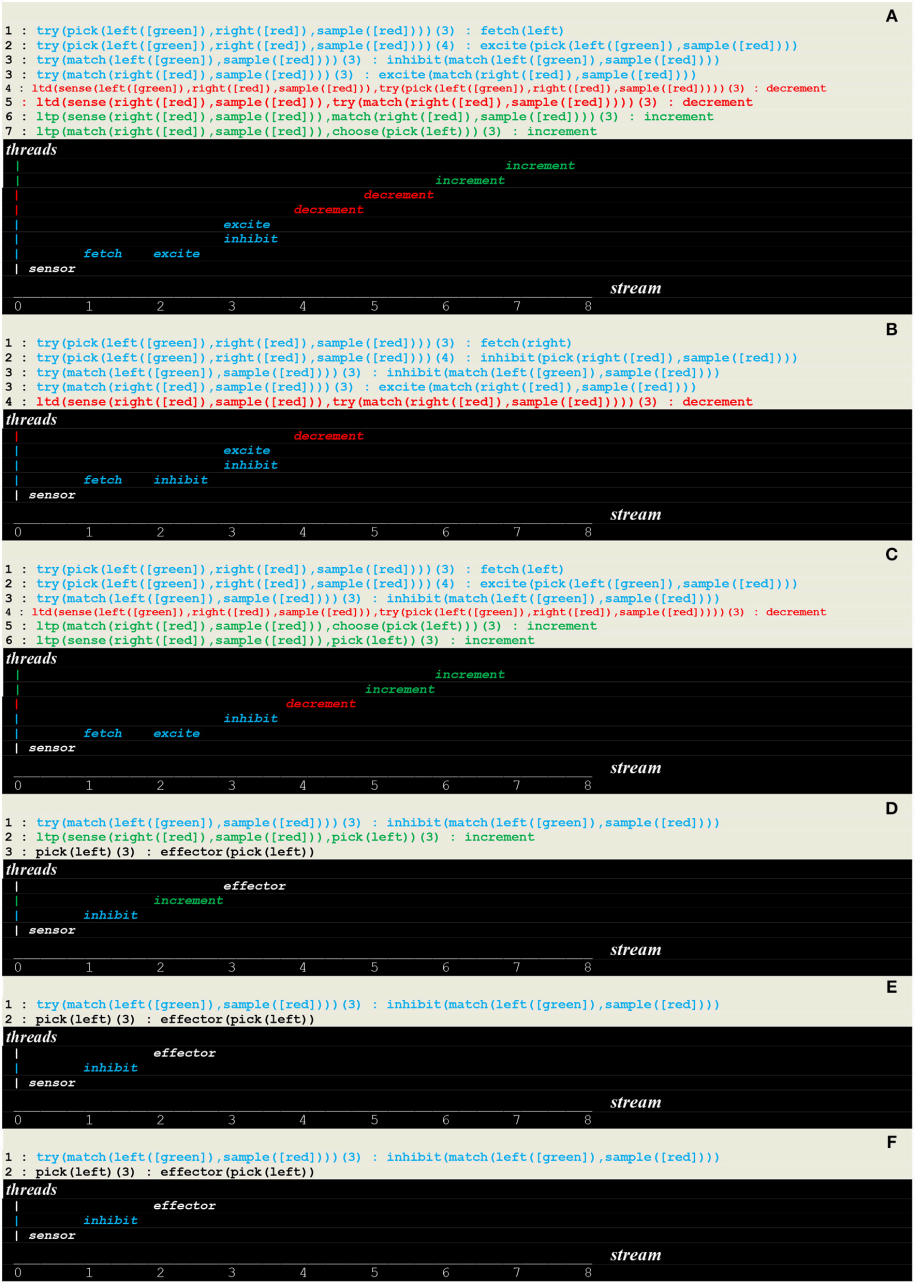
Execution trace. **(A–D)** Transient part. **(E,F)** Persistent part.

The same configuration i.e.,


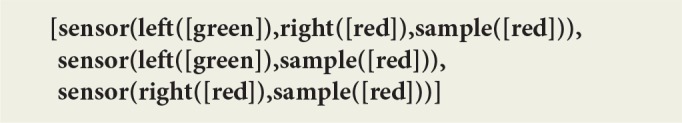


was repeatedly presented as input. The execution trace, which reflects the learning of a rule followed by its repeated application, contains a *transient* part (Figures 11A–D) made of successive internal stimuli followed by a *persistent* part (Figures 11E,F) presenting the recurrent pattern of a single internal stimulus i.e., inhibit, which signals the application of the rue commanding to “avoid matching the color.”

#### Simulating the Second and Third Level of Animal Awareness

Similar results have been obtained with simulations that were performed for experiments (Savage-Rumbaugh et al., 1980; Cole et al., 1982) characterizing, respectively, the second and third levels of animal consciousness. A subject with the second *level* is aware enough of a rule to transfer it across situations and thus to adopt for example a *win/stay lose/shift* rule. This implies a capacity to remember one's last choice, and has been implemented using a short term cache memory (*STM*) that allow for the modeling of a synchronized *recall* thread. The *third level* of animal awareness refers to the additional capacity to make a categorical judgment (e.g., to *sort* items) and has been implemented using an associative long term memory (*LTM*) that similarly allows for the *recall* of facts or events (see Bonzon, 2017 for details).

### Summary of Results

The results of the simulations presented above can be summarized as follows:

- as illustrated in Figure 9, unconscious reactive behaviors relying on configural learning produce *transient* traces that reflect the *asynchronous* processing of internal stimuli
- as illustrated in Figure 11, behaviors relying on a rule produce traces containing a *transient* part that reflect the *asynchronous* processing of internal stimuli, followed by a *persistent* part that present a recurrent *synchronous* pattern corresponding to the rule conscious application.

From these results, we postulate as a principle:

- ***persistent recurrent patterns in execution traces reveal potential synchronous roots of consciousness*.**

Let us recall from Figure 7 that our formalism relies on a fiber structure, where asynchronous *threads* having each their own *local time* are grouped into *streams*, which represent disjoints sets of simultaneously active threads, each stream being associated with a sequence number akin to a *global time*. The *synchronization* defined on this basis then simply associates the instruction being executed at thread time *T* with its supporting stream at sequence number *I*. Each recurrent pattern thus actually reflects the synchronization of an internal stimulus with its supporting stream e.g., in Figures 11E,F, the synchronization of stimulus

$$\begin{array}{l} \text{inhibit(match(left([green]),sample([red])))}\\ \text{in thread}\\ \text{try(match(left([green]),sample([red])))} \end{array}$$

at its local time (3) with its supporting stream at global time 1:

These recurring patterns constitute an example of emergent brain structures that might be associated with higher level cognitive capabilities. Following advances in the study of glial cells (Tadi et al., 2015; Dallérac and Rouach, 2016; Papouin et al., 2017), the possible relation of these synchronizations with consciousness could be found in the interaction between neurons and astrocytes. According to the “astrocentric hypothesis” (Robertson, 2013), conscious perception arises through a process of global synchrony in which information patterns carried by neuronal spike trains are transferred to astrocytic waves (Pereira and Furlan, 2009; Pereira et al., 2017). It is suggested that the persistent traces revealed in our simulation are at the roots of this transfer process.

## Discussion

This discussion will extend in three directions i.e., the hypothetical formal properties of the proposed formalism, its relevance to the study of consciousness, and its comparison with previous similar work.

### Hypothetical Formal Properties

The assessment of a system's formal properties should include both its *validation* and a theoretical account of its *computational power*. A throughout development of these two points is out of the scope of this paper. We shall therefore restrict ourselves to situate the proposed formalism within these contexts. Its various components are further presented below in the online Supplementary Information.

#### Validation

The validation of a system aims at providing a mathematical proof that its implementation satisfies its requirements i.e., that “*what it actually does*” is “*what it was designed to do*.” To try and answer the first question, let us consider the functional signature that can associated with the function representing the run of a model. The concept of a virtual machine that we use allows for emulating the execution of a program given in a *symbolic* language *S* on a system having its own *logical* language *L*. On the cognitive side, *virtual circuits*, which somehow correspond to cognitive software written in language *S*, are compiled into virtual code implications of language *L*. On its neural side, these implications are used in turn to deduce just in time instructions that get interpreted by the virtual machine i.e., this virtual machine actually performs *contextual deductions* (Bonzon, 1997). In addition, languages *I* and *O* define, respectively*, input/output* sentences captured by *sensors* and delivered to *effectors*. Running a model on a virtual machine then defines the function:

$$\begin{array}{l} run:\;I\;\times \;S\;\times \;\left(S\;\to \;L\right)\times L\;\to \;L\;\times \;O \end{array}$$

According to classical results in computer science, symbolic expressions that have been compiled and then interpreted by a virtual machine get their operational semantics from the transitions they induced on the state of this machine. In other words, what the system actually does is to update the virtual machine state. As there is no specified final state, whichever state the machine is in at any given time is acceptable and represents the simulated subject's current state of mind.

As for the second question (i.e., “what was this machine designed for”), the goal of the present work was to study the emergence of brain structures that might be associated with higher level cognitive capabilities i.e., with processes that are still unknown. In this perspective, the whole idea of validation and/or model checking, which eventually should to lead to ask “*how to specify a given task*,” may look premature.

#### Computational Power

Following the pioneering work of Siegelmann and Sontag (Siegelmann and Sontag, 1995), the computational equivalence between *rational recurrent neural networks* and *Turing machines* has become the starting point for the study of devices with *super Turing* computational power. Various extensions incorporating concepts such as *rational* vs. *analog* machine and *interactive* vs*. evolutive* machines have recently culminated in a new equivalence stating that “*basic neural models combining the two crucial features of evolvability and interactivity are actually capable of super-Turing computational capabilities, irrespective of whether their synaptic weights are modeled by rational or real numbers*” (Cabessa, 2012; Cabessa and Villa, 2013). In other words, taking into account both evolving and interactive capabilities in a neural net model provides an “alternative and equivalent way to the incorporation of the power of the continuum toward the achievement of super-Turing computational capabilities.” Intuitively, our own model, which incorporates both a mechanism of communication based on concurrent threads and an implementation of synaptic plasticity based on Hebbian learning, satisfies the conditions required to belong to the class of super Turing computational devices. The proof of this statement will eventually require a substantial effort toward mapping our formalism into the primitive operations that are allowed in such proofs.

### Relevance With the Study of Consciousness

Very generally, unconscious and conscious behavior have been described, respectively, as lacking conscious attention and as enjoying an introspective reporting capability (Shanahan, 2010). Various studies have focused on the search for the signature of the neural activity that differentiates between the two, but their overall results appear inconsistent (Dehaene and Changeux, 2011). Some of these results however are compatible with our postulate as stated in section Summary of Results. As an example, experiments related to a delayed matching to sample task (Dehaene et al., 2003) have suggested that the neural signature of unconscious vs. conscious perception could be a *local coordination* vs. a *global synchronization* of neural activity. Further results (Dehaene et al., 2006; Melloni et al., 2007) about the same task have concluded that *transient synchronization* is the critical event that triggers an access to consciousness. Our postulate is also consistent with the proposal (Lamme, 2003) of recurrent interactions, first *locally* within the visual system, and then *globally* into parieto-frontal regions, as well as with the hypothesis (Zeki, 2003) of an *asynchronous* construction of visual perception in distributed sites before binding into a “macro-consciousness.” By referring to synchronized events associating a stimulus with a sensory stream, it is also compatible with another approach (Morsella et al., 2015) concluding that the origin of consciousness could be found at the level of processing that is shared with “representations of the immediate external environment.” Hypothetically, as noted in section Summary of Results, persistent traces revealed in our simulations could be at the root of the transfer process from neural spike trains to astrocytic waves (Pereira and Furlan, 2009). Our concept of a virtual machine offering an interface between two domains (see section Bottom Up Design of Virtual Circuits of the Supplementary Information) could constitute the adequate tool for modeling such a transfer.

### Related Work

Previous work related to the modeling of brain and cognition using symbolic methods, and more generally to global brain simulations and the emergence of consciousness, are now reviewed.

In an extension of his early work on classical conditioning (Klopf, 1988), Klopf Johnson et al. (2001) did propose a computational model of learned avoidance that relies on an internal clock controlling both classically and instrumentally conditioned components, thus allowing for an explicit “proprioceptive feedback” i.e., a kind of primitive consciousness. This proposal opposed the then dominant paradigm requiring an evaluative feedback from the environment. This opposition did rest on the argument that “*animals do not receive error signals during learning*,” thus pointing out to the biological implausibility of error-correction back propagation i.e., an argument that, notwithstanding the proven effectiveness of this technique as a tool for functional approximation, is still valid today for brain research.

Using classical results on Hopfield networks and attractors (Hopfield, 1982), Balkenius and his co-workers (Balkenius et al., 2018) did implement a *memory model for robots*. In this model, a prototypal form of consciousness arises from sensory information filled in a memory that in turns produces memory transitions over time, thus creating an inner world that is used both to interpret external input and to support “thoughts disconnected from the present situation.” A far reaching but questionable conclusion of this study is that “an inner world is a *sine qua non* for consciousness.”

The work by Deco et al. (2008) falls in the category of “whole (or global) brain” simulations. Their theoretical account follows an overall *statistical* strategy. Degrees of freedom are successively reduced to resolve an otherwise intractable computational problem. Populations of spiking neurons get first reduced to distribution functions describing their probabilistic evolution, giving then rise to neural fields defined by differential operators involving both temporal and spatial terms. It finally proposes a measure for partitioning the brain into functionally relevant regions, this so-called “dynamical workspace of binding nodes” being supposedly responsible for binding information into conscious perceptions and memories. As in our own proposal, this formalism uses a multilevel architecture, which in this case distinguishes between the single neuron level, the mesoscopic describing how neural elements interact to yield emergent behavior, and the macroscopic level of dynamical large-scale neural systems such as cortical regions, the thalamus, etc. Each level of this description relates to neuroscience data, from single-unit recordings, through local field potentials to functional magnetic resonance imaging (fMRI). In conclusion, this formalism uses analytical and statistical tools to search for existing patterns in a functioning brain. In contrast, our own framework, which is constrained solely by a symbolic model of synaptic plasticity, proposes a tool for shaping the brain by linking perception to behavior trough a mechanism of hebbian learning.

Besold and Kühnberger (2015) envision a system that operates on different levels corresponding to the layers in a system's architecture in order to update network structures via the artificial equivalent of synaptic dynamics. Our formalism relying on a virtual machine can be considered as an attempt to implement this architecture via a conceptual abstraction of synaptic plasticity. Our formalism also bears some similarities with a new model of neural networks, namely *fibring neural networks* (Garcez and Gabbay, 2004) that, similarly to threads, allow for the activation of groups of neurons and thus represent different levels of abstraction.

With a few notable exceptions (e.g., Smith, 1992; Ruksénasz et al., 2009; Su et al., 2014), system validation is an issue that is seldom addressed in computational cognitive neuroscience. In order to obtain symbolic descriptions of neuronal behavior that allow for model checking, Su et al. have applied concurrency theory in a framework extending classical automata theory with communicating capabilities. A network of communicating automata is then mapped into a labeled transition system whose inference rules (for both internal transitions and automata synchronizations) define the semantics of the overall model. Su et al. further show that, in accordance with our own approach, asynchronous processing is not only a more biologically plausible way to model neural systems than do conventional artificial neural networks with synchronous updates, but also offers new perspectives for the cognitive modeling of higher level cognitive capabilities through emergent synchronous processes.

## Author Contributions

The author confirms being the sole contributor of this work and has approved it for publication.

### Conflict of Interest Statement

The author declares that the research was conducted in the absence of any commercial or financial relationships that could be construed as a potential conflict of interest.

## References

[B1] Ashby F. G. Helie S. (2011). A tutorial on computational cognitive neuroscience, modeling the neurodynamics of cognition. J. Math. Psychol. 55, 273–289. 10.1016/j.jmp.2011.04.003 21841845 PMC3153062

[B2] Baars B. A. (1988). Cognitive Theory of Consciousness. Cambridge, UK: Cambridge University Press.

[B3] Balkenius C. Tjøstheim T. A. Johansson B. Gärdenfors P. (2018). From focused thought to reveries: a memory system for a conscious robot. Front. Robot. AI 5:29 10.3389/frobt.2018.00029 PMC7805698 33500916

[B4] Besold T. Kühnberger K. (2015). Towards integrated neural–symbolic systems for human level AI: two research programs helping to bridge the gaps. Biol. Ins. Cogn. Arch. 14, 97–110. 10.1016/j.bica.2015.09.003

[B5] Bonzon P. (1997). “A reflective proof system for reasoning in contexts,” in Proc AAAI97. Available online at: www.aaai.org/Papers/AAAI/1997/AAAI97-061.pdf

[B6] Bonzon P. (2017). Towards neuro-inspired symbolic models of cognition: linking neural dynamics to behaviors through asynchronous communications. Cogn. Neurodyn. 11, 327–353. 10.1007/s11571-017-9435-3 28761554 PMC5509613

[B7] Buzsaki G. Llinas R. (2017). Space and time in the brain. Science 358, 482–485. 10.1126/science.aan8869 29074768 PMC5998813

[B8] Cabessa J. (2012). Interactive evolving recurrent neural networks are super-turing, in ICAART, eds Filipe J. Fred A. L. N. (Setubal: SciTePress), 328–333.

[B9] Cabessa J. Villa A. (2013). The super-turing computational power of interactive evolving recurrent neural networks. LNCS 8131, 58–56 10.1007/978-3-642-40728-4_8

[B10] Cole S. Hainsworth F. R. Kamil A. C. Mercier T. Wolf L. L. (1982). Spatial learning as an adaptation in hummingbirds. Science 217, 655–657. 10.1126/science.217.4560.655 17817537

[B11] Dallérac G. Rouach N. (2016). Astrocytes as new targets to improve cognitive functions. Prog. Neurobiol. 144, 48–67. 10.1016/j.pneurobio.2016.01.003 26969413

[B12] Deco G. Jirsa V. K. Robinson P. A. Breakspear M. Friston K. (2008). The dynamic brain: from spiking neurons to neural masses and cortical fields. PLoS Comput. Biol. 4:e1000092. 10.1371/journal.pcbi.1000092 18769680 PMC2519166

[B13] Dehaene S. Changeux J. P. (2011). Experimental and theoretical approaches to conscious processing. Neuron 70, 220–227. 10.1016/j.neuron.2011.03.018 21521609

[B14] Dehaene S. Changeux J. P. Naccache L. Sackur J. Sergent C. (2006). Conscious, preconscious, and subliminal processing: a testable taxonomy. Neuron 10, 204–211. 10.1016/j.tics.2006.03.007 16603406

[B15] Dehaene S. Naccache L. (2001). Towards a cognitive neuroscience of consciousness: basic evidence and a workspace framework. Cognition 79, 1–37. 10.1016/S0010-0277(00)00123-2 11164022

[B16] Dehaene S. Sergent C. Changeux J. P. (2003). A neuronal network model linking subjective reports and objective physiological data during conscious perception. Proc. Nat. Acad. Sci. U.S.A. 100, 8520–8525. 10.1073/pnas.1332574100 12829797 PMC166261

[B17] Frégnac Y. (2017). Big data and the industrialization of neuroscience: a safe roadmap for understanding the brain? Science 358, 470–477. 10.1126/science.aan8866 29074766

[B18] Frégnac Y. Bathellier B. (2015). Cortical correlates of low-level perception: from neural circuits to percepts. Neuron 88. 10.1016/j.neuron.2015.09.041 26447576

[B19] Garcez A. Gabbay D. (2004). Fibring neural networks, in Proceedings of the 19th Natl Confon Artificial Intelligence (AAAI Press).

[B20] Gross J. Schmitz F. Schnitzler I. Kessler K. Shapiro K. Hommel B.. (2004). Modulation of long-range neural synchrony reflects temporal limitations of visual attention in humans. Proc. Nat. Acad. Sci. U.S.A. 101, 13050–13055. 10.1073/pnas.0404944101 15328408 PMC516515

[B21] He K. Huertas M. Hong S. Z. Tie X. Hell J. W. Shouval H.. (2015). Distinct eligibility traces for LTP and LTD in cortical synapses. Neuron 88, 528–538. 10.1016/j.neuron.2015.09.037 26593091 PMC4660261

[B22] Hines M. L. Carnevale N. T. (1997). The NEURON simulation environnement. Neural Comput. 9, 1179–1209. 10.1162/neco.1997.9.6.1179 9248061

[B23] Hinton G. E. Osindero S. The Y. W. (2006). A fast learning algorithm for deep beliefs net. Neural Comput. 18, 1527–1554. 10.1162/neco.2006.18.7.1527 16764513

[B24] Hopfield J. J. (1982). Neural networks and physical systems with emergent collective computational abilities. Proc. Natl. Acad. Sci. U.S.A. 79, 2554–2558. 10.1073/pnas.79.8.2554 6953413 PMC346238

[B25] Huyck C. Passmore P. (2013). A review of cell assemblies. Biol. Cybern. 107, 263–288. 10.1007/s00422-013-0555-5 23559034

[B26] Johnson J. Li W. Li J. Klopf H. (2001). A computational model of learned avoidance behavior in a one-way avoidance experiment. Adapt. Behav. 9, 91–104. 10.1177/105971230200900205

[B27] Katz J. S. Bodily K. Wright A. A. (2008). Learning strategies in matching to sample: if-then and configural learning by pigeons. Behav. Proces. 77, 223–230. 10.1016/j.beproc.2007.10.011 18079071 PMC2290969

[B28] Klopf H. A. (1988). Neuronal model of classical conditioning. Psychobiology 16, 85–125.

[B29] Lamme V. (2003). Why visual attention and awareness are different. Trends Cogn. Sci. 7, 12–18. 10.1016/S1364-6613(02)00013-X 12517353

[B30] Letzkus J. Wolff S. Lüthi A. (2015). Disinhibition, a circuit mechanism for associative learning & memory. Neuron 88, 264–276. 10.1016/j.neuron.2015.09.024 26494276

[B31] Ma W. Pouget A. (2008). Linking neurons to behavior in multisensory perception: a computational review. Brain Res. 1242, 4–12. 10.1016/j.brainres.2008.04.082 18602905

[B32] Markram H. Muller E. Ramaswamy S. Reimann M. W. Abdellah M. Sanchez C. A.. (2015). Reconstruction & simulation of neocortical microcircuitry. Cell 163, 456–492. 10.1016/j.cell.2015.09.029 26451489

[B33] Marr D. (1982). Vision: A Computational Investigation into the Human Representation and Processing of Visual Information. New York, NY: Freeman.

[B34] Melloni L. Molina C. Pena M. Torres D. Singer W. Rodriguez E. (2007). Synchronization of neural activity across cortical areas correlates with conscious perception. J. Neurosci. 14, 2858–2865. 10.1523/JNEUROSCI.4623-06.2007 PMC6672558 17360907

[B35] Morsella E. Godwin C. Jantz T. Krieger S. Adam Gazzaley A. (2015). Homing in on consciousness in the nervous system: an action-based synthesis. Behav. Brain Sci. 39, 1–17. 10.1017/S0140525X15000643 26096599

[B36] Papouin T. Dunphy J. Tolman M. Foley J. C. Haydon P. G. (2017). Astrocytic control of synaptic function. Philos. Trans. R. Soc. B 372:20160154. 10.1098/rstb.2016.0154 28093548 PMC5247586

[B37] Pepperberg I. Lynn S. (2000). Possible levels of animal consciousness with reference to grey parrots (*Psittaccus erithacus*). *Am. Zool* 40, 893–901. 10.1093/icb/40.6.893

[B38] Pereira A. Foz B. Rocha A. (2017). The dynamical signature of conscious processing: From modality specific percepts to complex episodes. Psychol. Consci. Theor. Res. Prac. 4, 230–247. 10.1037/cns0000115

[B39] Pereira A. Furlan A. (2009). On the role of synchrony for neuron–astrocyte interactions and perceptual conscious processing. J. Biol. Phys. 35, 465–480. 10.1007/s10867-009-9147-y 19669426 PMC2750741

[B40] Robertson J. (2013). Astrocyte domains and the three-dimensional and seamless expression of consciousness and explicit memories. Med. Hypotheses 81, 1017–1024. 10.1016/j.mehy.2013.09.021 24099930

[B41] Ruksénasz R Back J. Curzon P. Blandford A. (2009). Verification-guided modeling of salience and cognitive load. Formal Aspects Comput. 21, 541–569. 10.1007/s00165-008-0102-7

[B42] Savage-Rumbaugh E. S. Rumbaugh D. M. Smith S. Lawson J. (1980). Reference, the linguistic essential. Nature 210, 922–925. 7434008 10.1126/science.7434008

[B43] Shanahan M. (2010). Embodiment and the Inner Life: Cognition and Consciousness in the Space of Possible Minds. Oxford: Oxford University Press.

[B44] Siegelmann H. Sontag E. (1995). On the computational power of neural nets. J. Comput. Syst. Sci. 50, 132–150. 10.1006/jcss.1995.1013

[B45] Singer W. (1993). Synchronization of cortical activity and its putative role in information processing and learning. Annu. Rev. Physiol. 55, 349–374. 10.1146/annurev.ph.55.030193.002025 8466179

[B46] Smith L. (1992). A framework for neural net specification. IEEE Trans. Softw. Eng. 18, 601–612. 10.1109/32.148478

[B47] Stern P. H. (2017). Neuroscience: in search for new concepts. Science 358, 464–465 10.1126/science.358.6362.464 29074764

[B48] Su L. Gomez R. Bowman S. (2014). Analysing neurobiological models using communicating automata. Formal Aspects Comput. 26, 1169–1204. 10.1007/s00165-014-0294-y

[B49] Tadi M. Allaman I. Lengacher S. Grenningloh G. Magistretti P. J. (2015). Learning-induced gene expression in the hippocampus reveals a role of neuron-astrocyte metabolic coupling in long term memory. PLoS ONE 10:e0141568. 10.1371/journal.pone.0141568 26513352 PMC4625956

[B50] van der Velde F. de Kamps M. (2015). The necessity of connection structures in neural models of variable binding. Cogn. Neurodyn. 9, 359–337. 10.1007/s11571-015-9331-7 26157510 PMC4491338

[B51] Wright A. A. (1997). Concept learning and learning strategies. Psychol. Sci. 8, 119–123 10.1111/j.1467-9280.1997.tb00693.x

[B52] Wright J. J. Bourke P. D. (2013). On the dynamics of cortical development: synchrony and synaptic self-organization. Front. Comput. Neurosci. 7:4. 10.3389/fncom.2013.00004 23596410 PMC3573321

[B53] Yarstev M. (2017). The emperor's new wardrobe: rebalancing diversity of animal models in neuroscience research. Science 358, 466–469. 10.1126/science.aan8865 29074765

[B54] Zagha E. Ge X. McCormick D. A. (2015). Competing neural ensembles in motor cortex gate goal-directed motor output. Neuron 88, 565–577. 10.1016/j.neuron.2015.09.044 26593093 PMC4660255

[B55] Zeki S. (2003). The disunity of consciousness. Trends Cogn. Sci. 7, 214–7218. 10.1016/S1364-6613(03)00081-0 12757823

[B56] Zeki S. (2015). A massively asynchronous, parallel brain. Philos. Tran. R. Soc. B 370:20140174. 10.1098/rstb.2014.0174 25823871 PMC4387515

[B57] Zentall T. Edwards C. Moore B. Hogan D. (1981). Identity: the basis for both matching and oddity learning in pigeons. J. Exp. Psychol. 7, 70–86. 10.1037/0097-7403.7.1.70

