# Supplementary material for: Symbolic Modeling of Asynchronous Neural Dynamics Reveals Potential Synchronous Roots for the Emergence of Awareness
Source: Front Comput Neurosci. 2019 Feb 12;13:1. doi: 10.3389/fncom.2019.00001 (PMC6380086; doi:10.3389/fncom.2019.00001)
Supplement: Supplementary file 1 [file Data_Sheet_1.docx]

**Supplementary information**

**1. Material and methods**

Our overall methodology can be described as follows:

a) *micro scale* virtual circuits implementing synaptic plasticity through asynchronous communicating processes are first defined

b) *meso scale* virtual circuits corresponding to basic cognitive processes are then composed out of these micro scale circuits

c) virtual circuits are finally compiled into *virtual code* to be interpreted by a virtual machine.

***1.1 Bottom up design of virtual circuits***

The basic units of processing used here are constituted by *threads*. In Computer science, a thread is a sequence of instructions that executes concurrently with other threads, may coexist with other threads to form a process and share resources such as memory. In the present context, a thread corresponds either to a single or to a group of neurons and will be represented by a symbolic expression enclosing an instruction *tree*. Two essential differences between neurons and threads allow for the later to represent groups of neurons that might be interleaved at a higher level i.e.,

- contrary to a neuron, a thread can be simultaneously a source and a recipient by maintaining parallel asynchronous communications
- contrary to traditional neuron models in which incoming signals are summed in some way into an integrated value, thread inputs can be processed individually.

Sets of threads linking perception and action can assembled to into functional unit called *fibers*. When activated by sensors, fibers constitute dynamical processes, called *streams,* which do correspond to active Hebbian cell assemblies^27^. Hebbian cell assemblies represent a theoretical framework that offers functional explanations of psychological phenomena by linking them to physiological processes. As such they do provide an interface between the neural and cognitive levels. In our formalism, streams are driven by a *virtual machine* that similarly functions as an interface. In computer science, the concept of a virtual machine*,* exemplified by the Java machine, constitutes the key mechanism that allows for interfacing software with its physical support i.e., hardware. The concept of a virtual machine that we use here basically allows for emulating the execution of a program in a *symbolic* language *S* on a system having its own *logical* language *L*. Such a machine functions thus as an interface which allows for defining mesoscale circuits independently of the way the microcircuits are actually implemented. In the context of a model of brain multi-level structures (NB in the sense of a functional hierarchy of entities such as neuron → neural assemblies → cognitive states →.. i.e., to be distinguished from a topological arrangement of neuronal layers, such as the visual cortical layers V1 → V2 → V4 → IT → ..), using a virtual machine means that low level physiological details can be ignored and models of neural computation formulated by relating input and output (i.e., perception and behavior) at a symbolic level. Streams driven by virtual machines thus represent a computational framework that, equally to cell assemblies, provides an interface between the neural and cognitive levels. On the symbolic cognitive side *S* of the machine considered here, threads assembled in fibers giving rise to streams can be represented by *virtual circuits* and compiled into virtual code implications of a logical language *L.* On its neural side *L*, these implications are used in turn to deduce just in time instructions that get interpreted by the virtual machine (i.e., this virtual machine actually performs contextual deductions). Mesoscale circuits thus somehow correspond to cognitive software running on top of a biological substrate. To illustrate this, we present two examples of virtual circuits modeling two cases of simple animal behaviors.

***1.2 A virtual circuit implementing classical conditioning***

As a general evolution principle, organisms devise and use “tricks” for their survival. The ability to evaluate a threat by learning predictive relationships e.g., by associating a noise and the presence of a predator, is an example of such tricks realized by *classical conditioning*. In a classical conditioning experiment, a light tactile conditioned stimulus **cs** elicits a weak defensive reflex, and a strong noxious unconditioned stimulus **us** produces a massive withdrawal reflex. After a few pairings of stimuli **cs** and **us,** where **cs** slightly precedes **us**, a stimulus **cs** alone triggers a significantly enhanced withdrawal reflex i.e., the organism has learned a new behavior. This can be represented by virtual circuit given in Fig. 1.

**sense(cs)-*->=>-**

**/|\ |**

**LTP +-motor(X)**

**| |**

**sense(us)-+->=>-**

**Figure** 1**.**  A mesoscale virtual circuit implementing classical conditioning.

In Fig. 1, the threads **sense(us)** and **sense(cs)** are coupled with sensors capturing external stimuli **us** and **cs** and correspond to sensory neurons. The thread **motor(X)**, where **X** is a parameter (or variable) that will be instantiated, is coupled with an effector and corresponsd to a motor neuron. Finally, the thread **ltp** (for *long term potentiation*) acts as a facilitatory interneuron reinforcing the pathway (i.e. augmenting its *weight*) between **sense(cs)** and **motor(cs)**. The communication protocols depicted by the symbol **->=>-** and **/|\** represent a synaptic transmission (i.e., the symbol **->=>-** stands for a *synapse*) and the modulation of a synapse, and the signs * and + correspond respectively to the conjunction of converging signals and either a choice of converging signals or the splitting of a diverging signal. Classical conditioning then follows from the application of hebbian learning i.e., “neurons that fire together wire together”. Though it is admitted today that classical conditioning in aplysia is mediated by multiple neuronal mechanisms including a postsynaptic retroaction on a presynaptic site, the important issue is that the learning of a new behavior requires a conjoint activity of multiple neurons. This activity in turn depends on the temporal pairing of the conditioned and unconditioned stimuli, which in conclusion leads to implement the thread **ltp** as a *detector of coincidence*.

***1.3 A virtual circuit implementing a simple case of operant conditioning***

The ability to assess and to remember the consequences of one's own actions is another example of associative learning providing survival advantages. In this case, operant conditioning associates an action and its result, which can be *positive* or *negative*. Toward this goal, the organism will receive either an *excite* or an *inhibit* internal stimulus (corresponding for instance to a reward or punishment) that will lead in turn to a reinforcement or a rejection of the action.

As an example of a simple thought experiment, a pigeon learning to discriminate between grains and pebbles receives two possible vectors **I** of external visual stimuli. The circuit given in Fig. 2, represents the interaction of four threads **sense(I)**, **try(accept(I))**, **accept(I)** and **reject(I)**, together with two threads **ltp** and two opposite threads **ltd (**for long term depression). In addition to the external stimuli, this circuit incorporates two internal stimuli **excite(accept(I))** and **inhibit(accept(I))** that correspond to feedbacks from probing the food according to a predefined set of accepted elements.

**---*->=>-accept(I)**

**| /|\**

**| LTP**

**| |**

**| +-----------------------------------**

**| | |**

**| LTD |**

**| \|/ |excite(pick(I))--**

**sense(I)-+---*->=>-try(pick(I))|**

**| /|\ |inhibit(pick(I))-**

**| LTD |**

**| | |**

**| +-----------------------------------**

**| |**

**| LTP**

**| \|/**

**---*->=>-reject(I)**

**Figure 2**  A meso scale virtual circuit implementing simple operant conditioning

At the beginning of the simulation, the pathways from **sense(I)** to **try(pick(I))** is open, while the pathways to both **accept(I)** and **reject(I)** are closed. After a few trials, the pigeon will have learned to close **try(pick(I))** and to open either **accept(I)** or **reject(I)**. This process matches a fundamental principle in circuit neuroscience according to which *inhibition* in neuronal networks during baseline conditions allows in turn for *disinhibition*, which then stands as a key mechanism for circuit plasticity, learning, and memory retrieval.

***1.4 Representing virtual circuits by symbolic expressions***

The circuit given in Fig. 1 gives rise to the following symbolic expression ***s*** *ϵ* ***S***:

**threads(aplysia(reflex)):**

**[thread(sense(us),**

**[fire(ltp(sense(cs),motor(cs))),**

**send(motor(us))]),**

**thread(sense(cs),**

**[merge(ltp(sense(cs),motor(cs))),**

**send(motor(cs))]),**

**thread(motor(X),**

**[receive(sense(X)),**

**effector(motor(X**))]),

**thread(ltp(Q,R),**

**[join(Q),**

**increment(weight(Q,R))])].**

In this example, the instruction tree of each thread reduces to a sequence of virtual instructions such as **fire**, **send**, **merge**, etc, but more generally an instruction tree can contain *alternatives* commanded by *guards.*

***1.5 Compiling instruction trees into virtual code implications***

Virtual code implications are compiled from thread expressions and have the form

*Guard => T:Instruction*

where *Instruction* is a virtual machine instruction, *T* its clock time, and *Guard* a logical expression defining a condition e.g., a synchronizing term. As an example, virtual code implications ***l*** *ϵ* ***L*** compiled from the above thread **sense(us)**are

**true => 1:fire(ltp(sense(cs),motor(cs)))**

**true => 2:send(motor(us))**

**true => 3:end**

In this simple example, successive clock time values (i.e., 1,2,3) correspond to a linear list traversal with no guards. More generally, this will give rise to a descent into trees containing guards e.g., **excite(pick(I))** guiding the descent into the tree of Fig. 2.

***1.6 Microcircuits implementing synaptic plasticity***

Virtual circuits rely on communication protocols that are pictured in thread diagrams by iconic symbols representing themselves microcircuits. These protocols can be defined by means of procedures that operate in pairs^24^

- synaptic transmission denoted by **->=>-** is implemented by a **send/receive** pair

- long term potentiation/depression *LTP/* is implemented by a **join/merge** and **link/meet** pairs

- short term cache memory *STM* denoted by **-<A>-** is implement by a **push/pull** pair

- associative memory *LTM*  based on long term storage and retrieval processes *LTS/LTR* and denoted by **-{P}-** is implemented by a **store/retrieve** pair.

**2. Top down construction of a virtual machine**

Let us consider a set of fibers together with sets of initial weights for pairs of communicating threads within fibers and sets of elements accepted by fibers. The virtual machine then consists of

- a set of registers comprising a local *clock* for each active thread, a sequence number (i.e. *seq*) for each stream*,* and four *internal* stimuli registers (i.e., *fetch, catch, excite, inhibit*)
- a set of local *signal* and *sync* queues attached to active threads
- a *content addressable memory* holding virtual code implications attached to threads, recorded transient synchronizations as well as sets of current weights and accept elements.

Let *Model* designate the state of the virtual machine. The machine itself functions as non deterministic learning automaton that operates on the constrained neural substrate represented by *Model*. Formally, it consists in repeating a *sense-react-reflect* cycle of embodied cognition defined by the following *run* procedure:

*run(Model)*

***loop*** *sense(Model)*

*react(Model)*

*reflect(Model)*

At the next level below, the *sense* procedure reflects the triggering of spike trains directed to sensory neurons. After possibly capturing an interrupt from sensors directed to a given active fiber, or *stream*, it updates *Model* using a transition function *input*:

*sense(Model)*

***if*** *interrupt(Stream(Input))*

***then***  *Model<− input(Model(Stream),Input)*

The function *inpu*t first terminates the interrupted stream by clearing all its registers and queues and then resets the clocks of the sensory threads associated with sensors. The *react* procedure consists of a loop calling on each active thread in any stream to first deduce a virtual machine instruction and then update *Model* using a transition function *output*  interpreting virtual machine instructions:

*react(Model)*

***for each*** *Stream(Thread),T:Instruction,*

***such that***  *ist(Model(Stream)(Thread),(clock(T), T:Instruction))*

***do*** *Model <− output(Model(Stream)(Thread), T:Instruction)*

Finally, reflecting consists in a loop calling on each *stream* at sequence sumber *I* to reflect the synchronization of a *thread* with a *stimulus*

*reflect(Model)*

***for each*** *Stream, I:Thread:Stimulus ,*

***such that***  *ist(Model(Stream),(seq(I), I:Thread:Stimulus))*

***do*** *report(Model(Stream), I:Thread:Stimulus)*

The *ist* predicate (standing for “is true”) implements *contextual* deduction^29^. Clock register values *T* are used to deduce, for each active thread, the next instruction satisfying the guard. Whenever a transition initiated by a thread succeeds, the thread clock is advanced and the next instruction is deduced and executed, and whenever it fails, the current instruction is executed again i.e., the transition is attempted until it eventually succeeds. Altogether, this amounts to descending into an instruction tree, with its local clock time corresponding to the currently reached depth. As postulated independently^3^, there is no central clock, leading thus to the modeling of the brain as a massively asynchronous, parallel organ.

***2.1 Virtual machine definition***

Operational Prolog specifications for the extended virtual machines and its instruction set. The definition of the basic threads implementing synaptic plasticity and memory are given at the end.

*NB Additions and modifications from the previously published specifications^24^ are underlined.*

Let *Interrupt*, the input sentence captured by *sensors* at successive run cycles, be represented by a list of the form

*[sensor(|X_1_),..sensor(|X_n_)]* .

An overall run is then defined as follows:

**run(Model) :- loop((sense(Model),react(Model),reflect(Model))).** *loop sense react reflect*

**sense(Model):- if(interrupt(Stream(Interrupt)),** *input interrupt*

**then((remove(Model(Stream)(_),clock(|_)),** *clear thread registers*

**remove(Model(Stream)(_),fetch(|_)),**

**remove(Model(Stream)(_),catch(|_)),**

**remove(Model(Stream)(_),excite(|_)),**

**remove(Model(Stream)(_),inhibit(|_)),**

**remove(Model(Stream)(_),signal(|_)),**

**remove(Model(Stream)(_),sync(|_)),**

**set(Model(Stream),seq(1)),** *reset stream sequence register*

**remove(Model(Stream),_:_:_)))),** *clear transient synchronizations*

**for_each(sensor(|X),** *for each sensor*

**such_that(member(sensor(|X),Interrupt)),**

**do(set(Model(Stream)(sense(|X)),clock(1))))))).** *fire sense thread*

**react(Model) :- for_each((Stream(Thread),T:Instruction),** *for each thread*

**such_that(ist(Model(Stream)(Thread),**

**(clock(T),T:Instruction))),** *retrieve instruction*

**do(Model(Stream)(Thread).(T:Instruction))).** *execute instruction*

**reflect(Model) :- for_each((Stream,I:Thread:Stimulus),** *for each stream*

**such_that(ist(Model(Stream,)**

**(seq(I),I:Thread:Stimulus))),** *retrieve synchronization*

**do(Model(Stream).(I:Thread:Stimulus))).** *report synchronization*

***2.2 Virtual machine instructions***

**Model(Stream)(P(|X)).(T:fire(Q(|Y))):-** *thread P(|X)* *fires thread Q(|Y)*

**T1 is T+1,**

**set(Model(Stream)(Q(|Y)),clock(1)),** *set Q(|Y) clock*

**set(Model(Stream)(P(|X)),clock(T1)).** *set P(|X) clock*

**Model(Stream)(P(|X)).(T:end):-** *thread P(|X)* *ends*

**remove(Model(Stream)(P(|X)),clock(T)).** *remove clock*

**Model(Stream)(P(|X)).(T:send(Q(|Y))):-** *thread P(|X)* *sends signal to thread Q(|Y)*

**T1 is T+1,**

**if_not(ist(Model(Stream)(Q(|Y)),clock(_)),** *receiver not active*

**then((set(Model(Stream)(Q(|Y)),clock(1))))),** *set receiver clock*

**if_not(ist(Model(Stream),weight(P(|X),Q(|Y))(W)),** *no attached weight*

**then(if(ist(Model(Stream),initial(P(|X),Q(|Y))(W)),** *declared weight*

**then(set(Model(Stream),weight(P(|X),Q(|Y))(W))),** *set declared weight*

**else(set(Model(Stream),weight(P(|X),Q(|Y))(0)))))),** *set inhibit weight*

**if_not(ist(Model(Stream)(P(|X)),signal(send(Q(|Y)))),** *no send signal*

**then(insert(Model(Stream)(P(|X)),signal(send(Q(|Y)))))),** *queue send signal*

**set(Model(Stream)(P(|X)),clock(T1)).** *set sender clock*

**Model(Stream)(Q(|Y)).(T:receive(P(|X))):-** *thread Q(|Y)* *receives signal from thread P(|X)*

**T1 is T+1,**

**if(ist(Model(Stream)(P(|X)),signal(send(Q(|Y)))),** *sender signal*

**then(if((ist(Model(Stream),weight(P(|X),Q(|Y))(K)),K>0),** *excite level*

**then(set(Model(Stream)(Q(|Y)),clock(T1)))))).** *set receiver clock*

**Model(Stream)(P(|X)).(T:merge(Q(|Y))):-** *thread P(|X)* *merges with thread Q(|Y)*

**T1 is T+1,**

**if_not(ist(Model(Stream)(P(|X)),signal(merge(Q(|Y))))** *no merge signal*

**then(insert(Model(Stream)(P(|X)),signal(merge(Q(|Y)))))),** *queue merge signal*

**set(Model(Stream)(P(|X)),clock(T1)).** *set clock*

**Model(Stream)(Q(|Y)).(T:join(P(|X))):-** *thread Q(|Y)* *joins thread P(|X)*

**T1 is T+1,**

**if(ist(Model(Stream)(P(|X)),signal(merge(Q(|Y)))),** *merge signal*

**then(set(Model(Stream)(Q(|Y)),clock(T1)))).** *set clock*

**Model(Stream)(P(|X)).(T:push(Q)):-** *push stm record Q*

**T1 is T+1,**

**remove(Model(Stream)(stm(_)),path),** *remove path to stm*

**remove(Model(Stream)(stm(_)),clock(_)),** *remove stm clock*

**set(Model(Stream)(stm(Q)),clock(1)),** *set stm clock*

**set(Model(Stream),weight(Q,stm(Q))(1)),** *set excite weight*

**set(Model(Stream)(P(|X)),clock(T1)) .** *set clock*

**Model(Stream)(P(|X)).(T:pull(Q)) :-** *pull stm record Q*

**T1 is T+1,**

**if(ist(Model(_)(stm(Q)),path),** *global path to* *stm*

**then(ist(Model(Stream),seq(I)),** *get stream sequence*

**insert(Model(Stream),I:P(|X):pull(Q)),** *record synchronization*

**set(Model(Stream)(P(|X)),clock(T1)))).** *set clock*

**Model(Stream)(lts(P(|X))).(T:store(P(|X))) :-** *store ltm record P(|X)*

**T1 is T+1,**

**if_not(ist(Model(Stream)(ltm(P(|X))),clock(_)),** *ltm(P|X) not active*

**then((set(Model(Stream)(ltm(P(|X))),clock(1)),** *set ltm clock*

**if_not(ist(Model(Stream),weight(P(|X),ltm(P(|X)))(W)),** *set inhibit weight*

**then(set(Model(Stream),weight(P(|X),ltm(P(|X)))(0))))))),**

**set(Model(Stream)(lts(P(|X))),clock(T1)).** *set lts clock*

**Model(Stream)(ltr(P(|X),Q(|Y),R(|Z))).(T:retrieve(P(|X))) :-** *retrieve from ltm*

**T1 is T+1,**

**if(ist(Model(_)(ltm(P(|X))),path),** *global* *path to ltm*

**then(ist(Model(Stream),seq(I)),** *get sequence*

**insert(Model(Stream),I:R(Z):retrieve(P(|X))),** *record synchronization*

**set(Model(Stream)(ltr(P(|X),Q(|Y),R(|Z))),clock(T1)))).** *set ltr clock*

**Model(Stream)(P(Q)).(T:feed(_)) :-** *feed path to P(Q)*

**T1 is T+1,**

**if((ist(Model(Stream),weight(Q,P(Q))(K)),K>0),** *excite weight*

**then((if_not(ist(Model(Stream)(P(Q)),path),** *no path*

**then(insert(Model(Stream)(P(Q)),path))),** *queue path*

**set(Model(Stream)(P(Q)),clock(T1))))).** *set clock*

**Model(Stream)(Thread).(T:increment(weight(P(|X),Q(|Y)))) :-** *increment weight*

**T1 is T+1,**

**if((ist(Model(Stream),weight(P(|X),Q(|Y))(W)),W<1),** *weight below threshold*

**then((W1 is W+1,** *increment weight*

**set(Model(Stream),weight(P(|X),Q(|Y))(W1))))),** *set weight*

**set(Model(Stream)(Thread),clock(T1)).** *set clock*

**Model(Stream)(Thread).(T:decrement(weight(P(|X),Q(|Y)))) :-** *decrement weight*

**T1 is T+1,**

**if((ist(Model(Stream),weight(P(|X),Q(|Y))(W)),W>0),** *weight above threshold*

**then((W1 is W-1,** *decrement weight*

**set(Model(Stream),weight(P(|X),Q(|Y))(W1))))),** *set weight*

**set(Model(Stream)(Thread),clock(T1)).** *set clock*

**Model(Stream)(Thread).(T:choice(X)):-** *random selection in list X*

**T1 is T+1,**

**random(R,X),** *random choice R*

**set(Model(Stream)(Thread),fetch(R)),** *set fetch stimulus*

**write(T:fetch(R)),nl,** *report asynchronous stimulus*

**ist(Model(Stream),seq(I)),** *get stream sequence*

**insert(Model(Stream),I:Thread:fetch(R)),** *record synchronization*

**set(Model(Stream)(Thread),clock(T1)).** *set clock*

**Model(Stream)(Thread).(T:test(Accept(|X))):-** *test accept element*

**T1 is T+1,**

**if(setof(Y,ist(Model(Stream),Accept(|Y)),List),** *list of accepted elements*

**then(if(member(X,List),** *element X in list*

**then(set(Model(Stream)(Thread),excite(Accept(|X))),** *set excite stimulus*

**write(T:excite(A(|X))),nl,** *report stimulus*

**ist(Model(Stream),seq(I)),** *get stream sequence*

**insert(Model(Stream),I:Thread:excite(A(|X))))),** *record synchronization*

**else(set(Model(Stream)(Thread),inhibit(Accept(|X))))** *set inhibit stimulus*

**write(T:inhibit(A(|X))),nl,** *report stimulus*

**ist(Model(Stream),seq(I)),** *get stream sequence*

**insert(Model(Stream),I:Thread:inhibit(A(|X)))))))),** *record synchrone*

**set(Model(Stream)(Thread),clock(T1)).** *set clock*

**Model(Stream)(Thread).(T:transmit(X|Y)):-** *noisy transmission of X conditional to Y*

**T1 is T+1,**

**random(R,[X,Y]),** *weighted random choice*

**write(T:catch(R)),nl,** *report asynchronous stimulus*

**ist(Model(Stream),seq(I)),** *get stream sequence*

**insert(Model(Stream),I:Thread:catch(R)),** *record synchronization*

**set(Model(Stream)(Thread),catch(R)),** *set catch stimulus*

**set(Model(Stream)(Thread),clock(T1)).** *set clock*

**Model(Stream)(Thread).(T:effector(P)):-** *virtual effector*

**T1 is T+1,**

**nl,write('>>'),write(effector(P)),nl,** *report effector*

**ist(Model(Stream),seq(I)),** *get stream sequence*

**insert(Model(Stream),I:Thread:effector(P)),** *record synchronization*

**set(Model(Stream)(Thread),clock(T1)).** *set clock*

**Model(Stream).(I:Thread:Stimulus)) :-**

**nl,write(I:Thread:Stimulus),nl,** *report synchronization*

**remove(Model(Stream),I:Thread:Stimulus),** *clear synchronization*

**I1 is I+1,** *increment stream sequence*

**set(Model(Stream),seq(I1))))).** *reset stream sequence*

***2.3 Basic threads implementing synaptic plasticity, memory and synchronization***

**threads(Model):**

**[thread(ltp(Q,R),** *long term potentiation*

**[join(Q),**

**increment(weight(Q,R))]),**

**thread(ltd(Q,R),** *long term depression*

**[join(Q),**

**decrement(weight(Q,R))]),**

**thread(lts(P),** *long term storage*

**[store(P),**

**increment(weight(P,ltm(P)))]),**

**thread(ltr(P,Q,R),** *long term retrieval*

**[retrieve(P),**

**increment(weight(Q,R))]),**

**thread(ltm(P),** *long term memory*

**[feed(_)]),**

**thread(stm(P),**  *short term memory*

**[feed(_)])].**
